# Our Bureau of Information

**Published:** 1912-03-30

**Authors:** 


### OUR BUREAU OF INFORMATION.

### Our Brotherhood of Service and the Strike.

Besides the miners themselves there are more than three-quarters of a million men idle in Britain at this time. The miners are at war with their employers, and indirectly with society as a whole, and if their wives and children suffer from the strike, they at least suffer in hope that present pains mean future profit. But the other workers who are stopped for want of coal to carry on their industry have no such consolation. The best they hope for is to start again with as little delay as possible. But they have no expectation of better wages or of greater security. The days in which they are idle, the privations which they undergo, are pure loss without a hope of compensation. Their need, their sufferings, make a strong claim on all who belong formally or informally to the brotherhood of service. At a time like this visits, which under different conditions might be resented, are welcomed, and friendly relations may be established between rich and poor which will continue long after the present crisis is past. It is a time for organised charity, and a time also for personal service. In homes where decent comfort prevailed there is now privation, and where there was before no more than a scant supply of necessaries, absolute destitution. We know what that means. It means the death of many young children; it means the weakening of the growing generation in a way that will tell upon them in after years. That it means the cutting off of many old people also is sad enough, but this has not the national importance of the other facts. But every death of a young person is a national loss. There are differences of opinion as to whether it is wise to help the children of those who are on strike. Those of sterner stuff argue that to assist the children of a striker is to make it possible for him to resist longer. But if, as seems to be the case, there are many among the strikers themselves who would go back to work if they were allowed by their leaders, this may be carrying logic too far. And in any case, at an exceptional time like this there is something to be said for the personal claims of each individual, if it be only a baby in arms, to be regarded as a separate entity. And among those about whose claims there can be no cavil there is much to do.

Many municipalities in districts where the usual industries are stopped by the strike are organising food supplies. At this Lenten season, when so many of the ordinary claims of social life are put aside, there must be time for those who will to give assistance or—what is equally necessary—inquiry into cases in connection with this work. Personal help of this sort, although the helper does not spend a penny out of his own pocket, may be of the greatest value. Indeed, it is often better to give what one can to the central organisation than to expend it in those doles which are so often the outcome of emotion rather than reason. Doubtless cases may arise where only the personal gift, offered delicately, will be accepted to meet the needs of the fireless hearth and the empty cupboard. We must each judge for ourselves what it is our duty to do. But the one thing certain is that all who have not yet been compelled to diminish their comforts must help those on whom the present industrial situation presses hard.

### Answers to Correspondents.

### School for a Deaf Girl.

Miss M. M., Portsmouth, asks : Will you kindly inform me if you know of any charitable institution into which a girl, who is deaf as a result of scarlatina, can be admitted and taught? The girl on whose behalf I am writing is bright and very intelligent, but, owing to her deafness, is unable to be taught with the other girls in school.

[Write to the British Asylum for Deaf and Dumb Females, Lower Clapton, London, N.E., or to the School for Oral Instruction of the Deaf and Dumb, 11 Fitzroy Square, London, W.C., or to the Homerton Residential School at Hackney. The Girls' Friendly Society also enrols deaf members, and the Young Women's Christian Association has a department for helping deaf girls, but its work is confined to those who have already been taught on the oral system.]

### Medical Scholarships for Women.

"Aspirant" asks : Are there any medical scholarships open to women?

[Yes, several. Of absolutely open scholarships there are the Beit Memorial fellowships and the Edith Pechey-Phipson scholarship, besides a Carnegie fellowship and a Carnegie scholarship. There are several scholarships in connection with the Royal Free Hospital, and most of the universities have medical scholarships open to their students.]

### Training of a Health Visitor.

"Sanitas" asks : What is the training necessary for a health visitor, and what is the salary?

[No special course of training has as yet been fixed for the work of a health visitor. Medical women, certificated nurses, trained midwives, and women holding the certificate of the Royal Sanitary Institute are preferred, but there is no definite standard. Salaries run from £65 to £100. In some places the woman sanitary inspector acts as health visitor also. In London there are fourteen health visitors, and about 220 in the provinces, but there is likely to be a greater demand in the future.]

### League of Mercy.

"Mercy" asks : Is there anything corresponding to the League of Mercy in the provinces?

[So far as we know, there is no organisation which exactly resembles the League of Mercy outside London and the home counties. But in many provincial towns there are Ladies' Auxiliaries which do much the same work—that is, collect small subscriptions for the benefit of the local hospitals. In many provincial hospital reports a list will be found of donations of half-a-crown and upwards, collected entirely by voluntary workers. With a little organisation on the part of the managers of hospitals and the natural social leaders in each district this work might be greatly enlarged.]

### Cancer Hospitals.

"C. R. N." asks : What hospitals are there for the treatment of cancer in the north of England?

[Consult Burdett's "Hospitals and Charities," published by the Scientific Press. Without more detailed information as to the locality you want, it is impossible for us to give specific information.]

### Typewriting and Eyesight.

"Typist" asks : Is typewriting bad for the eyes?

[It largely depends on the eyes. Most people can do typewriting without suffering. Others find that it causes pain, or raises a mist before the eyes. In such cases a doctor ought to be consulted. Sometimes the trouble can be cured by wearing suitable spectacles; if not, it will be necessary to give up the work.]

### Inspectors and Health Visitors.

"Puzzled" writes : What is the difference between a sanitary inspector and a health visitor? Is not their work the same?

[A sanitary inspector, who may be of either sex, has to see that certain Acts of Parliament affecting public health are complied with, and has the power to compel compliance by legal means. He may also insist on entering premises where he thinks these acts are being contravened. A health visitor is always a woman, and although appointed by a public authority has only the right to advise in various matters, and requires great patience and tact. Sanitary inspectors require to possess the certificates of the Sanitary Inspectors' Examination Board, but there is no special route to the profession of health visitor, though trained nurses and medical women are much esteemed in this capacity.]

### Rules for Correspondents.

Everyone who uses or wishes to help our Bureau of Information must be kind enough to observe the following rules:—

(1) Postal replies cannot be given. Exception will only be made in very special cases at the Editor's discretion.

(2) Queries or answers relating to different cases must be written, or preferably typed, on separate sheets of paper, and the writing must never be crossed.

(3) Questions and replies received not later than Wednesday will, as far as possible, be dealt with in our issue of the following Saturday week.

(4) Letters must have attached the name and address of the sender, with a pseudonym for publication if preferred.

(5) Applications referring to non-dependents where the need relates to business or employment must be accompanied by a postal order for 5s., when the requirement shall have publicity in these columns.

(6) All applicants for insertion in our list of Approved Homes must send a registration fee of five shillings. The omission of this leads to delay and gives avoidable trouble.

(7) *All communications* for this department *must be accompanied by the coupon* to be found at the bottom of the third page of the cover on the inside, and should be addressed to the Editor of the Bureau of Information, c/o The Hospital, 29 Southampton Street, Strand, London, W.C.

